# Comparison of serum vitamin D level in multiple sclerosis patients, their siblings, and healthy controls

**Published:** 2015-04-04

**Authors:** Ghazaleh Eskandari, Mahsa Ghajarzadeh, Mir Saeed Yekaninejad, Mohammad Ali Sahraian, Razieh Gorji, Faezeh Rajaei, Abbas Norouzi-Javidan, Alireza Faridar, Amirreza Azimi

**Affiliations:** 1 Brain and Spinal Injury Research Center, Neuroscience Institute, Tehran University of Medical Sciences, Tehran, Iran; 2 Department of Epidemiology and Biostatistics, School of Public Health, Tehran University of Medical Sciences, Tehran, Iran; 3 MS Research Center, Neuroscience Institute, Tehran University of Medical Sciences, Tehran, Iran; 4 Iranian Center of Neurological Research, Tehran University of Medical Sciences, Tehran, Iran; 5 Department of Neurology, Houston Methodist Hospital, Weill Cornell Medical College, Houston, TX

**Keywords:** Multiple Sclerosis, Vitamin D, Population Control, Siblings

## Abstract

**Background: **Multiple sclerosis (MS) is an autoimmune, neuro-inflammatory disease of central nervous system affecting physical, emotional, and cognitive aspects of patients. Association of vitamin D deficiency and MS has been shown in previous studies. The aim of this study was to evaluate serum vitamin D level in MS cases and their sex-matched healthy siblings (who are genetically near similar) and non-relative sex-matched healthy controls.

**Methods:** A total of 135 subjects enrolled in this case-control study. Group one (n = 45) consisted of patients with established MS. Group two (n = 45) included sex-matched healthy siblings of the group one and group three participants (n = 45) were non-relative sex-matched healthy controls. Demographic data (age, sex), level of education, daily sun exposure duration, and month of birth gathered for all. Serum sample of all participants was collected for 25-hydroxy vitamin D measurement.

**Results: **There was no significant difference between vitamin D level, sun exposure duration, education level, and season of birth in three evaluated groups. Mean vitamin D level was 8.2 ± 10.1 (nmol/l) in women and 13.3 ± 7 (nmol/l) in men (P = 0.001). There was a significant positive correlation between daily sun exposure duration and vitamin D level in whole participants (r = 0.28, P < 0.001) as well as in MS patients (r = 0.32, P = 0.030). Mean vitamin D level was significantly lower in participants who have born in spring and summer.

**Conclusion: **Vitamin D deficiency is high among Iranian population as well as MS patients.

## Introduction

Multiple sclerosis (MS) is an autoimmune, neuro-inflammatory disease of central nervous system affecting physical, emotional, and cognitive aspects of patients.^1^ It has been reported that more than two million individuals are affected all over the world, and annual incidence of the disease is increasing during the time in different geographical regions.^2^^-^^8^ In addition to strong genetic component, environmental factors such as vitamin D deficiency, Epstein-Barr virus infection, and smoking have been considered as influential factors in MS development.^9^

As MS prevalence is low in tropical areas and its incidence increases with distancing from the equator, it is hypothesized that latitude and sunlight have impacts on MS pathogenesis.^10^^-^^12^ In this regard, some previous studies reported that duration and intensity of sunlight as well as serum vitamin D level are negatively correlated with the incidence of MS.^13^^-^^15^ Pathophysiologically, vitamin D has a strong effect on cytokine profiles and plays a major role in modifying the inflammation in immune cells.^16^ In vitro studies support that vitamin D prevents interleukin (IL) 12, IL2 and interferon-gamma production along with B cells production inhibition.^16^^-^^18^ In addition, it has a preventive and therapeutic impact on TH17-mediated autoimmune diseases like MS.^19^

MS incidence is reported to increase rapidly in Tehran, Iran, in comparison with other cities all over the world.^8^ We hypothesized that vitamin D deficiency could be one of the underlying causes of increased MS incidence in our population.

In this regard, this study is designed to evaluate serum vitamin D level in MS cases and their sex-matched healthy siblings (who are genetically near similar) and non-relative sex-matched healthy controls to determine vitamin D levels in MS patients, their families, and healthy ones.

## Materials and Methods

This case-control study was approved by ethics committee of Tehran University of Medical Sciences and was conducted in the MS clinic of Sina Hospital (affiliated hospital of Tehran University of Medical Sciences). After filling informed consent forms, 135 participants were enrolled in three groups. Group one (n = 45) consisted of patients with established MS according to 2010 McDonald criteria.^20^ Group two (n = 45) included sex-matched healthy siblings of the group one, and group three participants (n = 45) were non-relative sex-matched healthy controls.

Inclusion criteria for group one were relapsing-remitting form of the disease, expanded disability status scale (EDSS) (EDSS which was assessed by an expert neurologist) score < 6, having a sex-matched healthy sibling with age range of maximum 10 years less or more and no other systemic diseases. Exclusion criteria were relapsing phase of the disease for MS patients and application of vitamin D supplement during last year for all participants.

Demographic data (age, sex), level of education, daily sun exposure duration (asked from each participant as a self-report question), and a month of birth gathered for all. Disease duration, medication, and Kurtzke EDSS score (after neurological examination by an expert neurologist) were recorded for each patient.

A volume of 2 ml blood sample between 20^th^ of March and 20^th^ of June was taken from all participants and then, 25-hydroxy vitamin D level was measured with chemiluminescent immunoassay method by using the DiaSorin LIAISON 25-OH Vitamin D Total assay in Masoud laboratory. 

According to endocrinology guideline,^21^ vitamin D level < 20 nmol/l considered as deficient and levels between 20-30 and 30-100 nmol/l considered as insufficient and sufficient, respectively. Subjects with lower than normal vitamin D levels were also subdivided into three subgroups with vitamin D level < 12.5 nmol/l, between 12.5 and 25 and the third group 25-30 nmol/l^22^ and the level of vitamin D deficiency was compared between MS group, siblings, and healthy controls.

All data were analyzed using SPSS software (version 18.0, SPSS Inc., Chicago, IL, USA). Continuous variables compared by means of independent sample t-test, Mann Whitney-U or ANOVA tests, and Fisher’s exact test was used to compare categorical variables. Correlation coefficient (Pearson or Spearman) calculated to assess the relationship between variables. Multiple regression analysis was used for the predictive value of age, education, and sun exposure duration for the vitamin D level. P < 0.050 was considered as significant.

## Results

One hundred and thirty-five participants enrolled in this study. Mean disease duration of patients was 2.5 ± 3.2 years and mean EDSS score was 1.3 ± 1.4. EDSS scores of male and female patients were not significantly different (male: 2.3 ± 1.2, female: 1.2 ± 1.4, P = 0.080).

There was no significant difference between vitamin D level, sun exposure duration, education level, and season of birth in three evaluated groups (Table 1). 

The severity of vitamin D deficiency was not significantly different between MS group versus siblings and healthy controls (Table 2).

Mean vitamin D level was 8.2 ± 10.1 (nmol/l) in women and 13.3 ± 7 (nmol/l) in men (P = 0.001). This mean value was significantly different between male and female ones in MS group (women = 8.7 ± 7.7, male = 15.9 ± 6.9, P = 0.040) while no significant differences was detected between male and female participants in two other groups. We did not find any significant difference in mean serum vitamin D levels between three study groups (P = 0.500). 

The rate of vitamin D deficiency was 86.6% in MS group and in sibling and healthy subject groups were, respectively, 84.4 and 93%. No significant difference found in vitamin D distribution in study groups by univariate analysis (P = 0.200).

There was no significant correlation between EDSS and vitamin D level and disease duration in patients (r = 0.09, P = 0.500 and r = 0.1, P = 0.500) whereas EDSS score was significantly correlated with age (r = 0.33, P = 0.020).

**Table 1:** Age, sun exposure duration, season of birth, and vitamin D level in three groups of participants

**Demographic characteristics**	**MS group**	**Siblings**	**Controls**	**P**
Age (mean ± SD)	30.3 ± 7.5	31.2 ± 8.7	31.1 ± 8.2	0.8
Education level (year) (mean ± SD)	13.8 ± 2.6	13.4 ± 3.1	14.5 ± 3.1	0.2
Daily sun exposure duration (min)	52.5 ± 41.4	60.0 ± 48.4	59.5 ± 36.5	0.6
Serum vitamin D level (nmol/l) (mean)	9.7 ± 7.9	9.4 ± 9.9	7.5 ± 11.6	0.5
Season of birth				0.3
Spring	19.0	11.0	11.0	
Summer	15.0	15.0	15.0	
Autumn	6.0	8.0	11.0	
Winter	5.0	11.0	8.0	
Median	6.5	6.0	4.2	
Vitamin D status				0.2
Deficient	39.0	38.0	42.0	
Insufficient	6.0	6.0	1.0	
Sufficient	0.0	1.0	2.0	

MS: Multiple sclerosis; SD: Standard deviation

**Table 2:** Level of vitamin D deficiency in three groups

**Vitamin D level**	**MS group**	**Siblings**	**Controls**	**P**
< 12.5 (nmol/l)	2	2		0.09
12.5-25( nmol/l)	14	8	5	
25-30 (nmol/l)	29	34	38	

MS: Multiple sclerosis

**Table 3:** Season of the birth and vitamin D level in participants

**Groups**	**Spring** **Number (mean ± SD)**	**Summer** **Number (mean ± SD)**	**Autumn** **Number (mean ± SD)**	**Winter** **Number (mean ± SD)**	**P**
MS	19 (8.9 ± 8.3)	15 (8.0 ± 7.3)	6 (15.3 ± 8.5)	5 (11.7 ± 6.1)	0.200
Siblings	11 (8.0 ± 8.5)	15 (8.3 ± 7.9)	8 (9.6 ± 9.1)	11 (12.3 ± 14.2)	0.700
Controls	11 (5.5 ± 5.6)	15 (4.8 ± 4.0)	11 (6.1 ± 6.6)	8 (17.4 ± 23.8)	0.060
Total	41 (7.7 ± 7.7)	45 (7.0 ± 6.7)	25 (9.4±8.4)	24 (13.9 ± 16.5)	0.030

MS: Multiple sclerosis; SD: Standard deviation

In MS group, 16 patients were under treatment by Avonex followed by Betaferon (6 cases) and Rebif (4 patients). Mean vitamin D levels were not significantly different in these treatment subgroups (13.4 ± 7, 6.8 ± 6.7, 11.2 ± 9, P = 0.300).

Although the season of the birth was not significantly different between case and controls (P = 0.300), mean vitamin D level was significantly lower in participants who have born in spring and summer (Table 3).

There was a significant positive correlation between daily sun exposure duration and vitamin D level in whole participants (r = 0.28, P < 0.001) as well as in MS patients (r = 0.32, P = 0.030) (Table 4).

**Table 4:** Linear regression considering vitamin D level as dependent variable and Sun exposure duration, education level, and age as independent variables

**Independent variables**	**B**	**P**
Sun exposure duration	0.29	0.010
Education level	0.01	0.800
Age	0.10	0.200

## Discussion

In this study, we evaluated the serum vitamin D levels in MS patients in comparison to their siblings as well as healthy controls. In addition to similar underlying genetics, patients and their siblings have grown up in similar environmental and socioeconomic states. It might help to limit the confounders that have affect in developing MS.

Our results did not show any difference in vitamin D level between MS patients and their siblings as well as healthy controls. It is in contrast to most previous studies that reported lower serum level of vitamin D in MS patients than healthy controls.^23^^-^^25^ However, in studies which conducted in Switzerland and Finland, the prevalence of vitamin D deficiency in MS patients was not lower than healthy ones in such countries.^26^^,^^27^

It is proposed that insignificant difference between serum vitamin D levels might be related to the fact that Tehran citizens generally have lower levels of serum vitamin D; a fact which makes it difficult to assess the significance of serum metabolite levels differences among different groups.^9^^,^^23^^,^^28^ To support this hypothesis, in a previous study, evaluating 1210 people in Tehran, 81.3% had vitamin D deficiency and prevalence of severe, moderate, and mild vitamin D deficiency was 9.5, 57.6, and 14.2 percent, respectively.^22^

Thus, in the presence of general vitamin D deficiency in our patients and controls, there is a possibility that other interacting factors including polymorphism in vitamin D receptor genes might play a key role in developing MS. However, existing epidemiological studies have insufficient power to address this hypothesis.^29^^,^^30^

Different factors such as insufficient sun exposure, clothing habits, air pollution, and insufficient intake of vitamin D are considered as effective factors in vitamin D deficiency.^31^ Although, Tehran, is located in 36° 21''N and has a mean sun exposure of 8 h per day, the high rate of air pollution in this city which prevents enough UV exposure to skin, could consider as a leading factor for vitamin D deficiency in people living in this city. In addition, significant higher level of vitamin D in men than women in this study could be indicative of clothing effect on vitamin D level. The mean sun exposure duration in participants of all three groups was near 1 h daily, and we found positive significant correlation between duration of sun exposure and vitamin D level in our population.

Although vitamin D has been known as calcium homeostasis modulator, its role as an environmental factor affecting MS prevalence becomes focus of interest in recent years.

Sunlight exposure, use of Vitamin D supplements, and higher levels of vitamin D in serum were associated with reduced risk of MS onset.^15^^,^^23^^,^^32^ On the other hand, literature show that higher vitamin D level was associated with lower relapse rate in MS cases along with findings which show that serum 25(OH) vitamin D was lower during relapse time in comparison with remission period in MS patients.^33^^-^^36^ These findings support immunomodulatory effects of vitamin D in autoimmune diseases. Although we found no statistically significant difference between vitamin D level and duration of sun exposure between three groups, sun exposure was positively correlated with vitamin D level and it considered as an independent predictor of serum vitamin D level.

Season of birth, according to exposure to ultraviolet radiation in early life, is important for developing diseases that affect central nervous system such as MS. In current study, we observed that most MS patients were born in spring in comparison with other two groups although the difference was not significant. In addition, vitamin D levels of participant who were born in spring and summer were significantly lower than other two groups. Willer et al. conducted a large population study, evaluating population of Canada, Great Britain, Denmark, and Sweden. They reported that people who born in May are at increased risk of MS in comparison with people born in the rest of the year especially in November.^37^

Our study had some limitations. First, our sample size was limited due to our inclusion criteria and time period of the study. Second, the study was single center study which was conducted in Tehran. In addition, the overall dietary vitamin D intake has not been evaluated in this study that might make a confounding bias in our findings. It is necessary to develop a large, multi-center study to evaluate vitamin D levels in MS patients.

## Conclusion

There are no significant differences in vitamin D levels between MS patients and their siblings as well as healthy controls. The prevalence of Vitamin D deficiency is very high in Iranian population.

## Notes:

**How to cite this article: **Eskandari G, Ghajarzadeh M, Yekaninejad MS, Sahraian MA, Gorji R, Rajaei F, et al. Comparison of serum vitamin D level in multiple sclerosis patients, their siblings, and healthy controls. Iran J Neurol 2015; 14(2): 81-5.
